# Multimodal Approaches to Patient Selection for Pancreas Cancer Surgery

**DOI:** 10.3390/curroncol31040167

**Published:** 2024-04-15

**Authors:** Hala Muaddi, LaDonna Kearse, Susanne Warner

**Affiliations:** Division of Hepatobiliary and Pancreas Surgery, Mayo Clinic, Rochester, MN 55902, USA; muaddi.hala@mayo.edu (H.M.);

**Keywords:** pancreatic ductal adenocarcinoma, neoadjuvant treatment

## Abstract

With an overall 5-year survival rate of 12%, pancreas ductal adenocarcinoma (PDAC) is an aggressive cancer that claims more than 50,000 patient lives each year in the United States alone. Even those few patients who undergo curative-intent resection with favorable pathology reports are likely to experience recurrence within the first two years after surgery and ultimately die from their cancer. We hypothesize that risk factors for these early recurrences can be identified with thorough preoperative staging, thus enabling proper patient selection for surgical resection and avoiding unnecessary harm. Herein, we review evidence supporting multidisciplinary and multimodality staging, comprehensive neoadjuvant treatment strategies, and optimal patient selection for curative-intent surgical resections. We further review data generated from our standardized approach at the Mayo Clinic and extrapolate to inform potential future investigations.

## 1. Introduction

**Figure 1:**
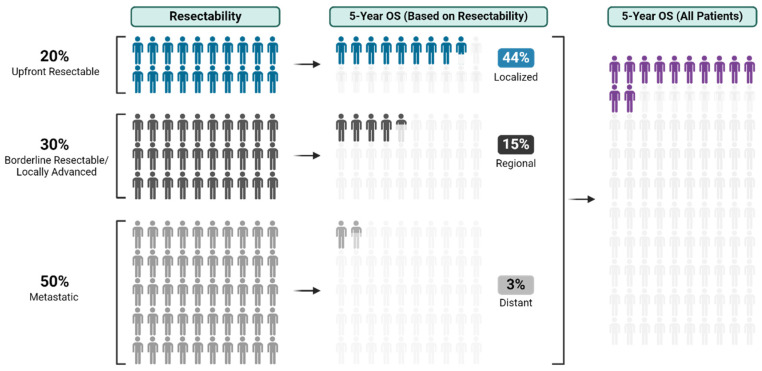
Resectability and survival of pancreatic ductal adenocarcinoma [1,2,3].

Pancreatic ductal adenocarcinoma (PDAC) presents formidable treatment challenges. As the third leading cause of cancer-related deaths in the United States, with a 5-year survival of 12%, PDAC warrants urgent exploration of novel treatment strategies (Figure 1) [1,2,3].

Surgical resection with pathologically negative margins (R0) remains a mainstay of potentially curative treatment for PDAC, with much lower survival observed in patients with microscopic (R1) or macroscopic (R2) residual tumor at resection [4,5]. Up to 50% of patients present with metastatic disease and are ineligible for curative resection (Figure 1) [6]. Of those presenting with localized PDAC, 15–20% have resectable disease without vascular involvement, and the remainder have borderline resectable or locally advanced disease, both of which present complex surgical management questions. True borderline resectable patients have up to an 80% chance of undertaking surgery, with 70% of those having R0 resections [7]. However, locally advanced patients have significantly lower chances of curative-intent R0 resection and should be counseled accordingly at treatment outset. While definitions of resectability vary widely [8,9,10,11,12,13], with thoughtful multidisciplinary multimodality neoadjuvant strategies, more and more locally advanced patients are undergoing curative-intent resections [14,15]. All that said, virtually all long-term survivors of PDAC receive both surgery and chemotherapy, making both modalities necessary to achieve cure [15,16].

This review explores recent advances in patient diagnosis, selection, and treatment. We also describe contemporary concepts of resectability and how these can be applied to carefully selected patients to optimize chances at R0 surgical resections and mitigate the risks of R1/2 resections of questionable oncologic benefit [17].

## 2. Pathology Acquisition and Biliary Drainage

PDAC presentation varies based on location; the majority (49.7−77.5%) originate in the pancreatic head, causing biliary obstruction and exocrine pancreatic insufficiency, while body and tail tumors present with nonspecific symptoms, more often at a later stage and with peritoneal dissemination [18,19,20]. When a mass is suspected on imaging, or when main pancreas ductal dilation is present without another definitive cause, referral for specialty imaging, pathological confirmation, and durable biliary drainage when appropriate are required to facilitate safe neoadjuvant therapy. 

Endoscopic ultrasonography (EUS) is integral for PDAC diagnosis and staging. It is most helpful for lesions < 2 cm and has sensitivity of up to 98%, whereas CT scan sensitivity can be limited to 65–75% [21,22,23]. Endoscopic retrograde cholangiopancreatography (ERCP) is the primary method for biliary decompression, with high success rates and a less than 5% complication rate at experienced centers [24]. At Mayo Clinic, when the presence of PDAC is known prior to ERCP, our preference is to place a short-covered, self-expandable metallic stent (SEMS) [25]. Patients with prolonged pre-drainage cholestasis will take time to normalize their bilirubin, which can delay initiation of neoadjuvant chemotherapy. At our institution, when bilirubin levels are >3.0 mg/dL, irinotecan is held, but mFOLFOX [oxaliplatin 85 mg/m^2^, leucovorin 400 (or levoleucovorin 200) mg/m^2^, and bolus 5-fluorouracil (5-FU) 400 mg/m^2^ on day one and continuous infusion of 5-FU 1200 mg/m^2^/d intravenously for the next 2 d (total 2400 mg/m^2^ over 48 h)] therapy will typically still be initiated.

Percutaneous transhepatic biliary drainage (PTBD) is reserved for patients in whom transpapillary drainage is not possible. The drain can remain external to decompress the biliary tree or can be internal–external to traverse the area of stricture into the duodenum. Ultimately, it can and should be converted into an internal drainage—ideally with a durable metal stent to optimize quality of life and avoid reintervention. Although PTBD is successful, it comes with a high reintervention and complication rate, ranging from 3–30% [26,27]. Comparative studies between PTBD and ERCP yield conflicting results; however, quality of life concerns with external drainage prompt most high volume centers to pursue multiple attempts at endoscopic interventions prior to engaging in percutaneous drainage [28].

While patients often arrive to our quaternary referral center with a myriad of different forms of biliary drainage and routes thereto, when patients are seen de novo at our institution prior to instrumentation with a suspected mass on CT, our preference is for them to undergo concurrent EUS with ERCP and, most typically, a short-covered metal stent. 

## 3. Complete Staging and Resectability Assessment

**Figure 2:**
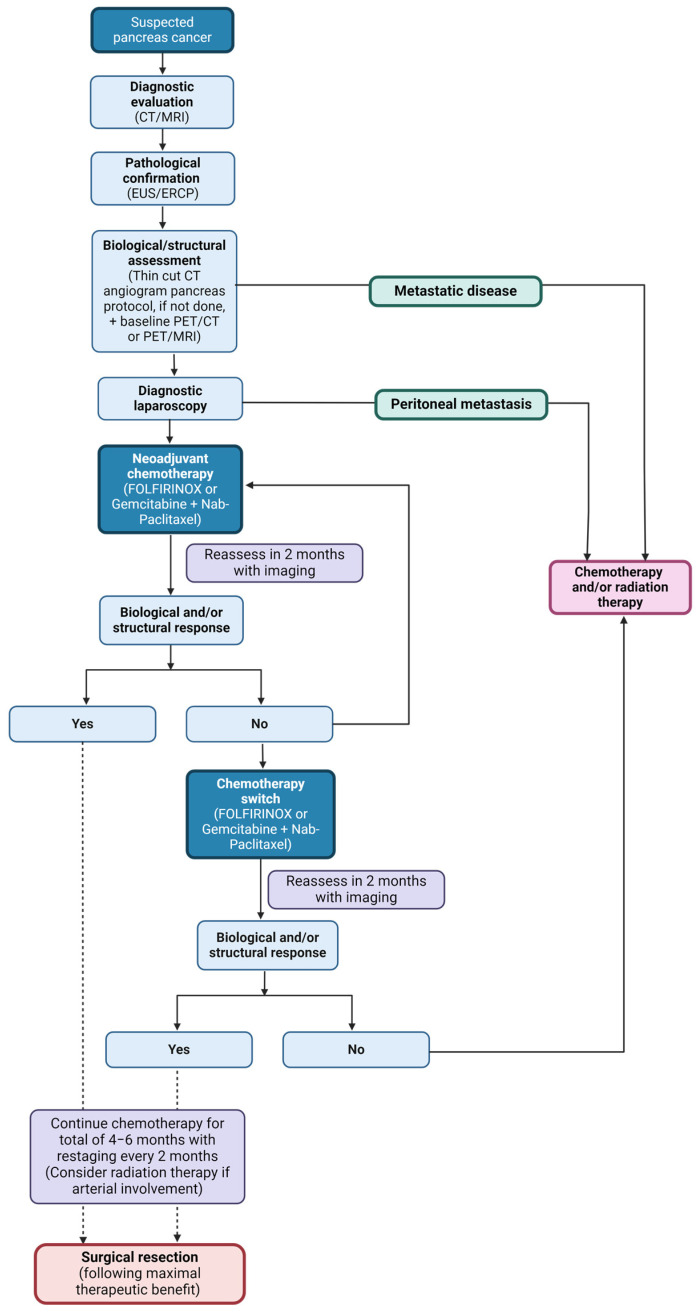
Diagnosis and management of PDAC. Outline of the diagnosis and management algorithm utilized at the Mayo Clinic. Once PDAC is suspected on diagnostic imaging, patients are required to undergo pathological confirmation with EUS and ERCP. Based on index of suspicion, patients simultaneously complete structural assessment using triple-phase pancreas protocol CT scan or MRI, in addition to complete staging with CT of chest and pelvis. This is done concurrently with biological assessment of serum CA 19-9 and PET scan. Prior to initiation of chemotherapy, patients undergo a diagnostic laparoscopy with washings to assess for occult disease. If no metastatic disease, neoadjuvant chemotherapy is initiated, generally with FOLFIRINOX, and patients are evaluated every 2 months to assess for biological and structural response and distant metastases. If no response or poor treatment tolerance, chemotherapy is switched to gemcitabine/nab-paclitaxel, and patients are reassessed in 2 months. In cases with no biological or structural response or with new metastatic disease, surgical resection is unlikely to be of benefit and patients are considered for additional chemotherapy, palliative radiation therapy, or clinical trials. In cases with good biological or structural response, patients continue this chemotherapy regimen until maximal response is achieved. Maximal response is demonstrated by stable disease on structural assessment and no or persistently low FDG-PET activity and/or CA 19-9 levels on biological assessment (4–6 months). In cases with vascular involvement, patients are considered for full-field chemoradiation prior to surgical resection.

For patients recently diagnosed with PDAC, a three-pronged approach is critical in the assessment of a resectability. This includes (1) structural assessment with axial imaging, (2) biological assessment with tumor markers and functional imaging, and (3) radiographically occult disease assessment with laparoscopy and washings. Figure 2 summarizes the standard diagnostic and treatment approach at our institute.

### 3.1. Structural Assessment

Liver metastasis, extensive vascular involvement, and peritoneal metastasis go undetected on initial staging imaging and are the primary reasons for aborting surgical resection. Therefore, structural assessment with high-quality axial imaging is paramount to appropriate staging and treatment planning. We contend that with adequate interpretation of high-quality and well-protocoled imaging, intraoperative “surprises” prompting aborted procedures should be extremely rare.

#### 3.1.1. Computed Tomography

Computed tomography (CT) scans, with a pancreas protocol, are the primary imaging modality for suspected pancreatic cancer, with a sensitivity of 89–97% [21]. While there are variations in imaging protocols across institutions, intravenous iodinated contrast and acquisition of thin slice scans (≤3 mm) with overlap and reconstruction are required. Two post-contrast phase acquisitions are crucial: the arterial phase (35–50 s), for delineating arterial anatomy and pancreatic parenchyma; and the portal venous phase (60–90 s), for venous anatomy and distant metastasis detection [29]. In addition, some centers may augment their protocol by providing negative oral contrast (e.g., water) to enhance duodenal wall examination [30]. Reformatted coronal and sagittal images for all phases aid in vascular anatomy assessment and in determining pancreatic lesion resectability. Our institutional practice is to obtain high-resolution CT Pancreas Protocol (triple phase with very thin <1.5 mm cuts) on all PDAC patients to assess resectability and vascular involvement, with templated reporting to enhance staging accuracy and standardize assessment of resectability [31]. 

#### 3.1.2. Magnetic Resonance Imaging

While CT scans are the preferred and more commonly used imaging modality, due to lower cost and widespread accessibility, high-quality magnetic resonance imaging (MRI) can add value when tumors are small and not detectable on CT scans, or when indeterminate liver findings are present on CT [32]. While not in routine use as part of resectability/structural assessment at our institution, MRI is commonly employed as part of functional imaging assessment with PET/MRI. It is most helpful for elucidating small abnormalities in the liver and peritoneum that might represent occult metastases.

### 3.2. Biological Assessment

#### 3.2.1. Blood Work

Patients suspected of having or diagnosed with PDAC require comprehensive blood work evaluation for baseline information and to assess biliary obstruction and nutritional status. A key component of the PDAC workup includes the tumor marker sialylated Lewis^a^ blood group antigen, commonly known as CA 19-9. In patients with PDAC and normal bilirubin, CA 19-9 can demonstrate sensitivity and specificity for PDAC ranging between 70–90% and can be highly correlated with resectability and survival rates [33,34]. However, it is imperative to recognize that the utility of CA 19-9 is significantly limited, as 10% of patients are non-secretors and up to one-third are normo-secretors with normal CA 19-9 levels despite aggressive disease [35,36]. The diagnostic value of CA 19-9 is further limited as it can be elevated due to certain medications, benign pancreaticobiliary diseases, and cholestasis.

Serial tracking of CA 19-9 during treatment is common in patients who present with elevated levels. A declining trend during neoadjuvant therapy correlates with improved survival [37], while elevated levels may indicate treatment failure, providing crucial insights into the effectiveness of the therapeutic approach [38]. Following surgical resection, normalization of CA 19-9 levels is expected, and persistently elevated levels indicate poor survival outcomes [39,40]. Therefore, CA 19-9 plays a crucial role in guiding treatment decisions and predicting patient outcomes.

#### 3.2.2. Positron Emission Tomography

**Figure 3:**
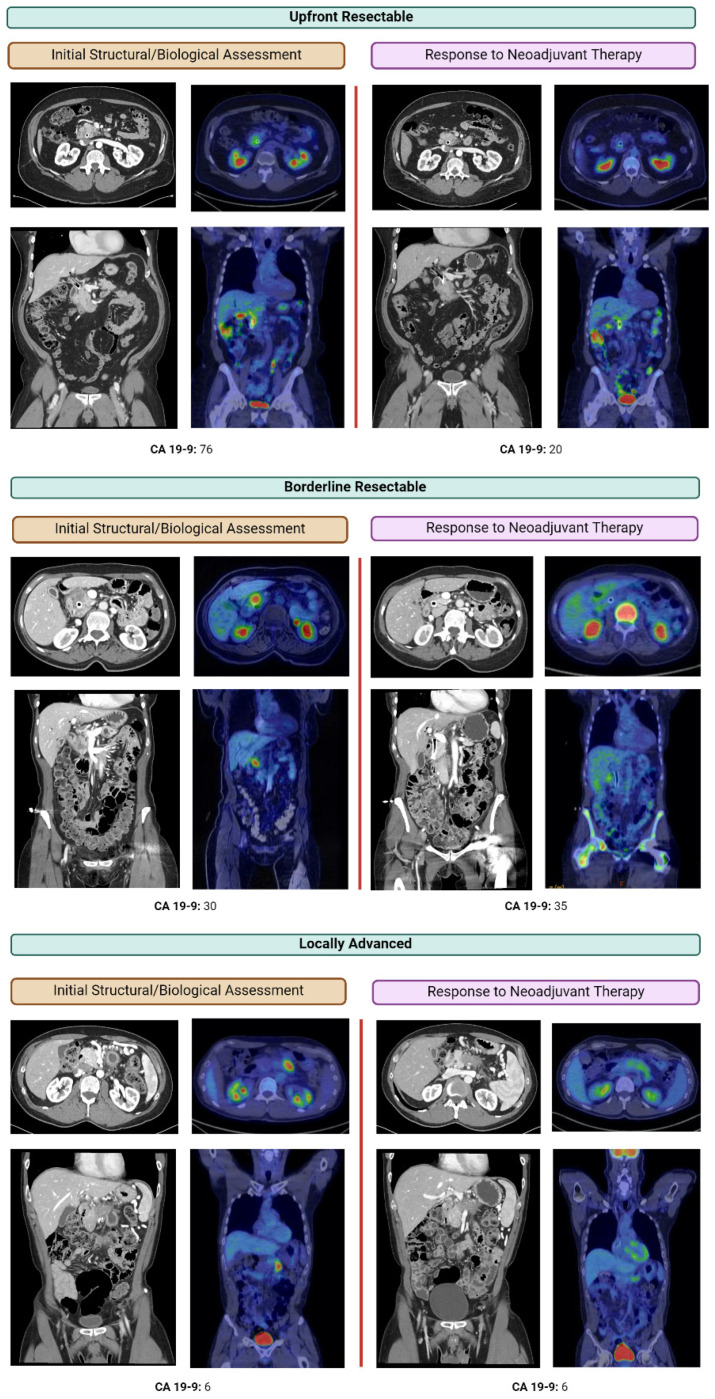
Structural and functional assessment of response to neoadjuvant chemotherapy using CA 19-9 and FDG-PET/CT or FDG-PET/MRI. CA 19-9 and FDG-PET/CT or FDG-PET/MRI before and after neoadjuvant chemotherapy for patients conventionally described as upfront resectable, borderline resectable, or locally advanced disease. Response to treatment is notable as a visual decrease in avidity of the tumor or calculation of SUVmax. FDG-PET scans are particularly helpful in assessment of treatment response of CA 19-9 non-secretors and normo-secretors.

Recent studies indicate that pathologic response rate, a surrogate marker for effective neoadjuvant chemotherapy, is the most significant predictor of survival [14,41]. This highlights the critical need for preoperative identification of patients who respond to neoadjuvant therapy [14,35,36,41,42,43]. Structural imaging like CT and MRI do not consistently predict pathologic response, and thus [^18^F]Fluoro-2-deoxyglucose-positron emission tomography (FDG-PET) can play an instrumental role in evaluating tumor response to chemotherapy (Figure 3) [42]. Indeed, many groups have correlated a decrease in standardized uptake value (SUVmax) on FDG-PET after neoadjuvant treatment with increased resectability, indicating SUVmax as a marker of response [44]. Our institution, among others, combines FDG-PET with CT or MRI to assess evidence of functional metabolic changes, which often precede the structural radiological changes seen on CT or MRI [45,46]. We use FDG-PET findings to ascertain whether the maximum benefit from neoadjuvant therapy has been achieved, thus enabling personalized decisions to extend for the duration of chemotherapy, including changing chemotherapy regimens [47], initiating chemoradiation [48], or recommending surgical intervention [14,17,49]. Conversely, the absence of an optimal FDG-PET response despite neoadjuvant chemotherapy raises concerns of chemoresistance, implying suboptimal postoperative survival, with or without resection [46]. In such instances, patients are appropriately counselled, and the risks of planned operations are carefully weighed against the predicted survival benefit, further exemplifying the crucial role of PET in tailoring treatment strategies for PDAC [46,47]. While not all institutions have the capacity to support serial PET imaging during neoadjuvant treatment, it is of particular importance in assessing response to treatment and should be highly considered in all patients, especially in the subset of patients who are non-secretors and normo-secretors [35,36,43,46].

### 3.3. Radiographically Occult Disease Assessment

#### Diagnostic Laparoscopy

To identify occult metastatic disease and prevent both unnecessary operative morbidity and unnecessary emotional duress, inherent to enduring 4–6 months of neoadjuvant treatment only to learn of stage IV disease, we routinely perform diagnostic laparoscopy with peritoneal lavage after PDAC diagnosis [50]. In our recent series of over 1000 patients undergoing diagnostic laparoscopy for presumed non-metastatic PDAC, diagnostic laparoscopy revealed gross metastatic disease or positive peritoneal washings in 18%. Importantly, 42% with positive cytology lacked grossly visible metastatic disease at time of laparoscopy [50]. This highlights the importance of washings and should dissuade less thorough practices of diagnostic laparoscopy on the same date as major resection. When we sub-analyzed these patients based on whether or not they had already received any neoadjuvant therapy, we were not surprised to find that chemotherapy decreases yield of laparoscopy and washings, with 23% of treatment-naïve patients found to have either gross peritoneal disease or positive peritoneal washings vs. 19% of those who had already received treatment. Thus, this intervention is paramount to both optimization of patient perioperative psychological safety and operating room utilization. Notably, analysis of peritoneal washings at our institution often includes the detection of CA 19-9 and CEA levels, with elevated levels more frequently observed in patients with positive disease identified on diagnostic laparoscopy [50]. Risk factors for positive diagnostic laparoscopy included younger age, distal pancreatic tumors, larger tumors with borderline or locally advanced disease, elevated serum CA 19-9 (≥35 U/mL), and elevated peritoneal CEA [50]. These findings, and others [51,52], underscore the significance of diagnostic laparoscopy in refining the staging process and guiding appropriate treatment strategies for patients with PDAC.

## 4. Neoadjuvant Treatment

The majority of PDAC long-term survivors have received both chemotherapy and curative-intent surgery. When given prior to resection, up to 80% of patients will receive all or most of their prescribed chemotherapy, whereas, when administered post-operatively, only 50% of patients will receive even 1–2 chemotherapy doses because of the associated post-operative morbidity [53]. In addition to ensuring adequate chemotherapy dosing, neoadjuvant chemotherapy offers advantages like assessing disease biology, identifying rapidly progressing tumors, enhancing negative margins, increasing the likelihood of node negative resections, and treating occult micro-metastatic disease [53,54].

While neoadjuvant chemotherapy is a mainstay of treatment for patients with borderline resectable and locally advanced tumors, wherein the rate of “conversion” to resectability ranges from 20–80% depending upon initial tumor involvement [53], the utility of neoadjuvant therapy for resectable disease in the available literature is less clear [55]. The majority of studies investigating neoadjuvant chemotherapy for resectable PDAC disease, until recently, were retrospective in design [14,54,56]. The PREOPANC-1 trial indicated a trend towards longer survival with gemcitabine-combined neoadjuvant radiotherapy compared to upfront surgery; however, this did not reach statistical significance [57]. Limitations of this trial include the use of a gemcitabine-based chemo-radiotherapy regimen that is no longer considered the standard of practice [58]. The NORPACT-1 trial exclusively included patients with resectable disease and concluded that there was no benefit to neoadjuvant FOLFIRINOX [59]. The major criticism of this work is the small number of planned neoadjuvant cycles and the lower number of patients (40%) completing all four cycles, which make it challenging to fully understand the role of neoadjuvant FOLFIRINOX. The PREOPANC-2 trial focused on comparing two neoadjuvant therapy regimens: total neoadjuvant FOLFIRINOX and neoadjuvant gemcitabine with radiotherapy followed by adjuvant gemcitabine [60]. There was no difference in survival benefit, resection rates, or adverse events between the two treatment groups. These trials, like others before them, showed increased adherence to a prescribed regimen in the neoadjuvant group. Future trials in progress aim to address some of the ongoing equipoises [61,62,63]. 

At Mayo Clinic, we adopted a standardized approach where, with rare exceptions, all patients with PDAC receive neoadjuvant therapy following resectability assessment and staging (Figure 2). The number of cycles can be tailored to PDAC resectability, such that patients with resectable disease are recommended to complete at least eight cycles or 4 months of uninterrupted treatment, whereas patients with locally advanced disease receive twelve cycles [14]. Patients with borderline resectable disease typically receive a range in between these two, the length of which is determined by individual factors such as structural, functional, and biochemical assessments of tumor response, alongside patient functional status and treatment toxicity. While receiving neoadjuvant therapy, patients are seen in follow up approximately every 2 months, with restaging imaging including a high-resolution CT pancreas protocol, CT chest, CT pelvis, FDG-PET/CT or FDG-PET/MRI, and CA 19-9 to assess for structural and functional imaging responses alongside biochemical tumor marker response. Following chemotherapy, selected patients receive long-course chemoradiation between 50–50.4 Gy, delivered in 25–28 fractions over 3 weeks and targeting the tumor, nodal basin, and involved vessels [14,64]. Those patients receiving neoadjuvant chemotherapy alone will typically go to resection within 3–6 weeks of the last chemotherapy infusion, whereas those undertaking chemoradiation will do so 3 weeks following chemotherapy completion, and will then go to surgery 4–8 weeks after completion of chemo-radiotherapy [14]. 

To derive maximum benefit from neoadjuvant therapy, patients must have ongoing treatment tolerance alongside measurable treatment response. For patient counseling, it is important to note that FOLFIRINOX has an approximate 75% rate of grade 3 adverse events, whereas gemcitabine/nab-paclitaxel has an approximate 50% rate of grade 3 adverse events [65]. Extrapolating from data in the metastatic setting suggesting enhanced efficacy of FOLFIRINOX compared to gemcitabine-based regimens, our practice is to offer FOLFIRINOX to those patients who can tolerate it [66]. At Mayo Clinic, our practice is to assess for structural and biological response every two months (after four cycles of FOLFIRINOX) in addition to assessing for tolerability and toxicity during each cycle (Figure 2). In circumstances where either treatment tolerance is compromised or when tumor progression or other unfavorable characteristics are identified at restaging, we switch to a different chemotherapy regimen and then re-stage in two months [47]. Unfavorable characteristics include a rise in CA 19-9 levels or SUVmax on PET/CT or PET/MRI imaging [47]. From our experience, PET imaging is superior in predicting pathologic response probability than CA19-9 and is more often used to guide treatment decisions [46]. In our recently reported experience, approximately 30% undergo a chemotherapy switch due to ineffectiveness or intolerance [47]. Of those, 72% achieve therapeutic benefit after the switch and proceed to curative-intent resection. Notably, there are no discernible differences in oncologic outcomes between patients switching chemotherapy and those continuing with the first-line regimen. Thus, there is no downside to chemotherapy switch in patients who are intolerant of first-line chemotherapy, or in whom initial tumor responses are unfavorable [47]. In our experience, chemo-switch enables personalized treatment while simultaneously enabling the testing of biology to preserve curative-intent treatment options for as many patients as possible [47].

## 5. Resectability

Different classification systems exist to categorize PDAC (Figure 3) [8,9,10,11,12,13]. Classically, PDAC is considered clearly resectable if the tumor shows no contact with the celiac trunk, hepatic artery, superior mesenteric artery (SMA), superior mesenteric vein (SMV), or portal vein. Borderline resectable tumors may abut the celiac artery, common hepatic artery, or SMA at less than 180 degrees, and may involve or deform the portal vein or the superior mesenteric vein (SMV) to an extent that still allows for resection and reconstruction [9]. Locally advanced disease includes patients with more extensive vascular involvement, such as celiac or SMA encasement > 180 degrees, encasement of the common hepatic artery up to or past the bifurcation of the hepatic arteries, and/or long segment vein involvement without reconstructible proximal or distal targets [9].

Pre-treatment classifications of resectability are critical to predicting the likelihood of R0 resection, which was historically considered the most important factor in determining patient outcomes and survival following resection [11,67]. The prognostic importance of this “curative” resection has been recently challenged by demonstrating comparable overall survival of patients undergoing R1 resection vs. those undergoing R0 resection [68]. These findings are further supported by randomized controlled trials, which did not show any difference in overall survival between R0 and R1 resections after receipt of adjuvant chemotherapy [69,70]. We also note that definitions of R0 vs. R1 vary internationally, which further complicates how we interpret the importance of this variable. While these evolving data suggest that long-term survival may be possible even in the context of R1 resections, at present, R0 resection should be the goal and most available evidence suggests that, if a margin positive resection is likely, that consolidative chemoradiation does less patient harm [5,14,71].

R0 resection can be challenging if the tumor is adjacent to vascular structures. However, experienced pancreatic surgeons increasingly incorporate vascular resection, particularly of the superior mesenteric vein and portal vein, when required to achieve R0 resection [10,72,73]. Survival following standard pancreatectomy is equivalent to pancreatectomy with R0 venous resection, however, this comes with the increased morbidity risk associated with venous reconstruction [74,75,76,77]. As a result, experts recommend confining venous resection to higher-volume centers with expertise in both pancreatic surgery and vascular reconstruction. 

With the neoadjuvant systemic and local therapy advances, experts now recognize a subgroup of patients who may benefit from pancreatectomy with en bloc arterial resection as well [17]. Patients who demonstrate a biological response to neoadjuvant therapy, via normalization of CA 19-9 and absent PET avidity, may proceed to definitive surgical resection with en bloc arterial resection [14]. Arterial divestment has been described as an alternative approach to arterial resection and reconstruction. This includes dissection and removal of the arterial sub-adventitia without arterial resection [78,79]. However, this surgical technique continues to be a point of debate, as the evidence for this technique is in its early stages and carries all of the surgical risks with the potential of an R1 margin [78]. Both are considered complex procedures and have significantly higher risks than standard pancreatectomy, with a 10–12% 90-day mortality [17,80,81]. Therefore, surgical resections for patients with arterial involvement should not be treated as first-line management, and resection should occur once the maximal effect of neoadjuvant treatment is achieved [17]. In these carefully selected patients and with the right technical expertise at high-volume centers, complex arterial reconstructions have improved survival when compared to palliative management [17,82,83,84]. 

The indications and use of neoadjuvant therapy vary among institutions. At the Mayo clinic, independent of vascular involvement, neoadjuvant therapy is typically recommended for all patients. Our studies have shown a remarkable 3-year overall survival of 59% and a median overall survival of 51.1 months for patients with borderline resectable and locally advanced PDAC who received neoadjuvant therapy [14]. In contrast, reported overall survival with use of neoadjuvant therapy from other institutions shows a median overall survival of 31.5 months [85]. This is notably more promising than a 3-year overall survival of 23% and a median overall survival of 15.3 months when neoadjuvant therapy is less structured [86]. These variations in outcomes may be attributed to differences in the number of chemotherapy cycles, the chemoradiation protocols, and the variable definitions of vascular involvement. Caution should be exercised when comparing outcomes across different institutions due to other unadjusted and unknown confounders. 

## 6. Conclusions

Several advancements have been made in the management of pancreatic ductal adenocarcinoma. A key aspect is the management and selection of patients who would benefit from and should proceed to definitive surgical resection. The increased utilization of neoadjuvant therapy has prompted a reevaluation of the traditional definitions and perceptions of what is considered resectable. Originally, the classical definitions of resectability were designed to guide preoperative treatment strategy for standard pancreatectomy. With the current advances in neoadjuvant therapy and en bloc surgical techniques, these traditional definitions may not accurately indicate inoperability or poor oncological outcomes. Thus, in summary and as previously described [17], in patients with locally advanced or borderline resectable tumors, we utilize the following guiding principles to determine whether patients would benefit from advanced procedures and the treatments described above: **responsivity, reconstructability, and recoverability** [17]. 

**Responsivity** is measured by structural assessment with cross-sectional imaging, biological assessment with functional imaging and biochemical assessment (CA 19-9), and occult disease assessment through diagnostic laparoscopy washings. Patients with non-metastatic disease are offered neoadjuvant chemotherapy with reassessment of response after 2–3 cycles. In cases with no response or poor tolerability to treatment, we advocate for chemotherapy switch [47]. The duration of neoadjuvant treatment is guided by the response of the disease, such that the objective of the extended duration of neoadjuvant chemotherapy, for cases with major vascular reconstruction, is to achieve complete or near-complete biochemical and functional response prior to initiating locoregional therapy followed by resection [14,17].

**Reconstructability** is less standardized and varies by surgeon experience and learning curve. This tenet requires assessment of tumor proximity to vascular structures and the ability to perform en bloc resection with R0 margins and restore gastrointestinal perfusion and continuity, venous outflow, and biliary drainage. Detailing the operative approach for vascular resection and reconstruction is imperative in the preoperative setting, as initial exploration during surgical resection alongside attempts to “peel off” tumors from vascular structures can lead to catastrophic bleeding and markedly inferior patient outcomes [17].

**Recoverability** refers to the patient’s preoperative fitness, comorbidities, perioperative morbidity and postoperative quality of life, and survival compared with nonoperative approaches [14,17]. Although not primarily addressed in this review, we would be remiss to neglect the importance of collaboration with medical colleagues in nutrition, endocrinology, infectious diseases, and rehabilitation to facilitate patients’ postoperative recovery and improve quality of life. At times, these issues may be the only hindrance preventing the patient from an operative procedure, as the surgical outcome may be more detrimental to their quality of life.
